# Comparison of Two Types of Overoxidized PEDOT Films and Their Application in Sensor Fabrication

**DOI:** 10.3390/s17030628

**Published:** 2017-03-19

**Authors:** Yun Hui, Chao Bian, Jinfen Wang, Jianhua Tong, Shanhong Xia

**Affiliations:** 1 State Key Laboratory of Transducer Technology, Institute of Electronics Chinese Academy of Sciences, Beijing 100190, China; huiyunccc@126.com (Y.H.); cbian@mail.ie.ac.cn (C.B.); jhtong@mail.ie.ac.cn (J.T.); 2 School of Electronic, Electrical, and Communication Engineering, University of Chinese Academy of Sciences, Beijing 100190, China; 3 CAS Center for Excellence in Nanoscience, National Center for Nanoscience and Technology, Beijing 100190, China; wangjinfen@nanoctr.cn

**Keywords:** overoxidized PEDOT, Au microelectrode, EIS, SEM, FTIR, oxygenic functional groups, roughness

## Abstract

Poly(3,4-ethylenedioxythiophene) (PEDOT) films were prepared by electro-oxidation on Au microelectrodes in an aqueous solution. Electrolyte solutions and polymerization parameters were optimized prior to overoxidation. The effect of overoxidation time has been optimized by cyclic voltammetry (CV) and electrochemical impedance spectroscopy (EIS), which results in the film overoxidized for 45 s at 1.35 V presenting a strong adsorption. The other one-step overoxidation film prepared by direct CV ranging from −0.6 V to 1.35 V was polymerized for comparison. Scanning electron microscope (SEM) analysis and Fourier transform infrared (FTIR) spectroscopy were used for monitoring morphological changes and the evolution of functional groups. Both of them indicate increased abundant oxygen functional groups and roughness, yet the products exhibit dendritic morphology and piles of spherical protrusions, respectively. Moreover, double-step overoxidized film showed better electrochemical performance toward lead ion sensing. These characterizations highlight some novel properties that may be beneficial for specific sensing applications.

## 1. Introduction

Over recent decades, conducting polymers, usually including polypyrrole (PPy), polyaniline (PANI), polythiophene (PTh) and their derivatives, have been extensively surveyed in the field of electroanalytical chemistry, due to their enhanced electrochemical performance for biochemical sensing [1,2]. As an important polythiophene derivative, poly(3,4-ethylenedioxythiophene) (PEDOT) features low oxidation potential, high electrical conductivity, good electrochemical activity, high stability and excellent biocompatibility, thus it was gradually applied to develop diverse biochemical sensors [3,4,5] and even neural probes [6].

PEDOT can be easily fabricated using both chemical oxidation and electropolymerization processes, either in the presence of high concentrations of powerful oxidants or under proper potentials. In terms of electropolymerization, EDOT can be doped with many anions, ranging from macromolecular polyanions such as poly(styrene sulfonate) (PSS) to small ones like perchlorate (ClO_4_^−^) [7].

Reports on overoxidized conductive polymers [8,9], have attracted great attention from researchers, particularly in the field of chemically modified electrodes. Li et al. suggested that overoxidized PPy (PPyox) film displayed remarkable cation exchange and molecular sieve properties along with porous structures [10]. During the last three years, some literature, albeit limited, has also demonstrated that PEDOT in an overoxidation state showed useful properties for analytical applications. Overoxidized PEDOT film-modified electrodes exhibited unique sensitivity and selectivity for the determination of dopamine and uric acid, successfully achieving the detection of submicromolar concentrations [11,12]. As for the affinity towards uric acid, overoxidized PEDOT exhibited nearly seven-times higher affinity than that of PPyox under the same preparation conditions [12], probably due to the higher conductivity of overoxidized PEDOT than PPyox. However, the mechanism for the obtained selectivity of overoxidized PEDOT films is not entirely clear. Lin et al. concluded that overoxidized PEDOT has the advantage of remarkable adsorption characteristics and recognition of inorganic and organic chemicals by contrast with non-overoxidized PEDOT [11]. To a large extent, this is attributed to the increase in hydrophobicity, abundant oxygen groups and a strong adsorption features for inorganic and organic cations [11].

As an irreversible degradation reaction, electrochemical overoxidation of PEDOT have been studied in many aspects recently. In [13], in situ ESR spectroelectrochemistry was performed to investigate the behaviour of charge-carrying species. Overoxidation also consists of three stages accompanied by the substitution of nucleophiles on the 3rd or 4th position of the thiophene units, the formation of carbonyls in the 2nd, 3rd and 5th positions and finally the formation of terminal carboxylic (COOH) groups [14].

Furthermore the experimental conditions have a great impact on the characteristics of the obtained products, including pH of the overoxidation medium [14] and different potentials [15]. Ujvári et al. have conducted a series of studies on overoxidation of electrodeposited PEDOT films [16,17,18]. They pointed out that increasing potential cycles resulted in morphological changes and structure evolution, particularly ones indicating a gradual improvement of crystallinity [16]. In the latest review [17], several experimental techniques suitable for monitoring the degradation process and overoxidation mechanisms proposed in the literature have been summarized.

However, not much work has been done focusing on the effect of overoxidation methods—one-step or double-step—on the electrochemical and morphological properties of PEDOT film-modified Au microelectrodes. The present paper concentrated on a comparison of the preparation and characterization of two different overoxidized PEDOT films. In order to gain a deeper insight into these issues, scanning electron microscopy, cyclic voltammetry, electrochemical impedance spectroscopy and Fourier transform infrared spectra have been employed. Moreover, PEDOT has been extensively studied for its application in sensor fabrication. Nael et al. have proposed PEDOT: PSS modified graphite carbon electrodes for the ultrasensitive detection of Pb^2+^ by chrono-amperometry [19]. Lead ion is considered to be a highly toxic heavy metal ion owing to its adverse effects on both health and environment, so its monitoring in environmental and biological samples is of vital importance for. In order to further test the sensing performance of the three types of PEDOT films, lead ion was selected as the analyte, and their electrochemical response to lead ion was tested and compared.

## 2. Material and Experimental

### 2.1. Reagents and Apparatus

3,4-Ethylenedioxythiophene (EDOT), Nafion (5 wt%), Tetrabutylammonium perruthenate (TBAP), were purchased from Sigma-Aldrich (St. Louis, MO, USA). AZ1500 and SU-8 were obtained from AZ Electronic Materials (Luxembourg, Luxembourg) and MicroChem Corp Company (Westborough, MA, USA), respectively. Lead standard stock solution was purchased from the China National Research Centre (Beijing, China). Lithium perchlorate, sodium dodecylsulfate (SDS) and sodium dodecyl sulfonate and all other chemicals were of analytical grade and were used without further purification. Deionized water with a resistivity of 18 MΩ·cm obtained from a Direct-Q 3 UV system (Merck Millipore Co., Billerica, MA, USA) was used for preparing the desired solutions and also for washing the electrodes and containers of the solutions throughout the experiments.

The optical microscopy image was carried out on a BX51 instrument (Olympus, Tokyo, Japan) using a 1.25× Olympus objective lens and 10× eye lens. SEM analysis was carried out on an S-4800 field emission scanning electron microscope (FE-SEM) from Hitachi (Tokyo, Japan). Fourier Transform Infrared (FTIR) spectroscopy was performed on a Spectrum One instrument (Perkin Elmer, MA, USA) with a diamond ATR accessory. Commercial Ag/AgCl (saturated KCl) served as reference electrode. The potential values reported are referred to such a reference electrode. All electrochemical measurements, including linear sweep voltammetry (LSV), cyclic voltammetry (CV), chronoamperometry, electrochemical impedance spectroscopy (EIS) and differential pulse stripping voltammetry (DPSV) were performed with the Reference 600 workstation (Gamry Instruments Co., Ltd., Warminster, PA, USA) by a three-electrode system consisting of the fabricated Au microelectrode and a Ag/AgCl reference electrode.

### 2.2. Microelectrode Fabrication

**Figure 1:**
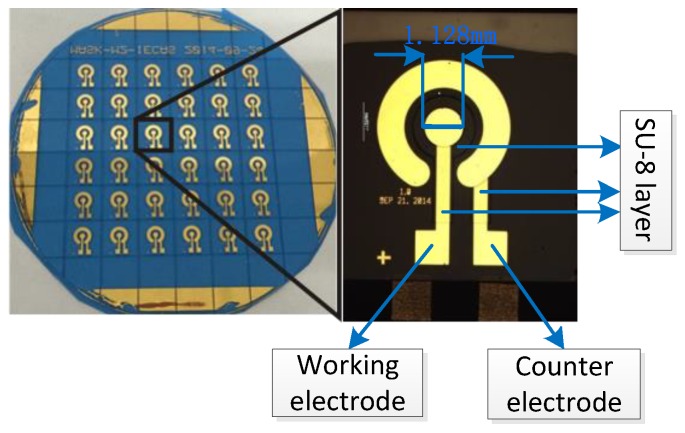
The pattern and structure under a 12.5× optical microscope of the Au microelectrodes.

The microelectrode chip was fabricated by a standard microelectromechanical systems (MEMS) technique in our lab. The two benefits of this approach are as follows: (1) a great number of identical electrodes are fabricated in a single batch; (2) the geometries of the electrodes can be accurately controlled at the same time. The metal line and electrodes were defined by means of lift-off of a layer stack of 200 Å Ta (adherent layer) and 2000 Å gold. An Au disk working electrode and an Au counter electrode compose the concentric structure, as shown in Figure 1. The Au disk working electrode exhibits a fixed area of 1 mm^2^ using SU-8 negative photoresist as insulting layer. Eventually the wafer was cut into 36 slices with a size of 8 mm × 8 mm, then the microelectrodes were wire-bonded and encapsulated on print circuit board strips. For one thing, the microelectrodes could be fabricated in batches, so it was economic and reproducible to some degree; for another, the microelectrodes displayed prominent advantages such as the lower iR drop, the faster mass transport rate, the higher signal-to-noise ratio and the larger current density.

### 2.3. Solutions and PEDOT Films Preparation

Prior to the surface modification, the microelectrode was physically cleaned by oxygen plasma etching with 50 W power for 4 min and then chemically cleaned in 0.5 M H_2_SO_4_ solution in the potential range of 0 to 1.5 V by CV continuously until a reproducible voltammogram characteristic of the gold electrode was obtained. Generally at the same scanning speed, similar reduction peak area, namely the amount of charge, signifies approximate active area of numerous Au electrodes from which we choose to do a series of comparison tests.

Three types of PEDOT films in different oxidized stage were prepared. Firstly LSV of 0.01 M EDOT in different electrolyte solutions were performed; secondly films with different polymerization cycles and potential range from −0.6 V to 1.0 V were prepared (denoted as PEDOT). Finally different overoxidation times at 1.35 V were imposed on deposited PEDOT layers in deoxygenated 0.1 M PBS (pH = 7.4) (denoted as double-step overoxidized PEDOT) and also film with the same cycles yet a potential ranging from −0.6 V to 1.35 V was prepared for comparison (denoted as one-step overoxidized PEDOT).

### 2.4. Characterization

The characterizations of PEDOT modified microelectrodes were performed by SEM, CV, EIS and FTIR. The common redox probe, 0.1 M KCl solution containing 5 mM [Fe(CN)_6_]^4−/3−^, was adopted for CV as well as EIS measurement. CV measurements were carried out from −0.2 to 0.6 V versus Ag/AgCl reference electrode. In regard to EIS measurements, the frequency ranges from 10^5^ to 0.5 Hz with the direct current potential set 0.26 V [20] and the alternating current potential set 5 mV. To facilitate the FTIR measurements, gold covered glass plate rather than microelectrode chip act as the working electrode. Gold plates can be obtained by directly sputtering Au on glass substrate and dicing it.

### 2.5. Electrochemical Response to Lead Ion

The electrochemical responses of the three types of PEDOT film-modified Au microelectrodes were recorded in 0.1 M HCl solution in the presence of 2.4 µM Pb^2+^ with DPSV, since stripping voltammetry provides an cost-effective way for simple and sensitive determination of heavy metals at low concentration level [21]. Parameters were set as follows: accumulation potential −0.9 V, accumulation time 120 s, pulse size 50 mV, scan rate 10 mV/s.

## 3. Results and Discussion

### 3.1. The LSV of 0.01 M EDOT in Different Electrolyte Solutions

**Figure 2:**
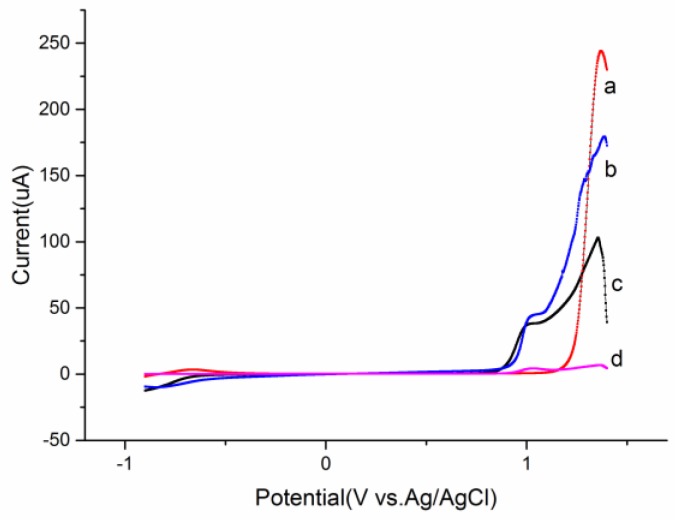
The LSV of 0.01 M EDOT in different electrolyte solutions: 0.01 M EDOT and 0.1 M TBAP in acetonitrile (a) 0.01 M EDOT in water (b) 0.01 M EDOT, 0.1 M LiClO_4_ and 5 mM C_12_H_25_SO_4_Na in water (c) and 0.01 M EDOT, 0.1 M LiClO_4_ and 5 mM C_12_H_25_SO_3_Na in water (d).

In order to select a proper electrolyte solution for overoxidation, 0.01 M EDOT was electropolymerized in four different solutions by LSV in the range of −0.9 V to 1.45 V. Previously numerous reports [12,22] have indicated that both the solvent and counter-ions have a dramatic influence on the electrical properties and morphology of the resulting polymers. As common anionic surfactants, sodium dodecyl sulfate and sodium dodecyl sulfonate, could not only lower the oxidation potential of EDOT, but also increase its solubility in aqueous and significantly improve the interface interaction between working electrode and solvent. As shown in Figure 2, the highest oxidation potential measured in acetonitrile means it is minimally affected by overoxidation. A solution containing 0.01 M EDOT, 0.1 M LiClO_4_ and 5 mM sodium dodecyl sulfate was chosen as the final electrolyte solution due to its lowest oxidation potential at 0.98 V and overoxidation potential at 1.35 V with a higher current response than in solution containing dodecyl sulfonate. In this paper, double-step overoxidation consists of initial deposition with the potential near 0.98 V and subsequent overoxidation at 1.35 V over different times.

### 3.2. The CV of Different Polymerization Potentials and Cycles

**Figure 3:**
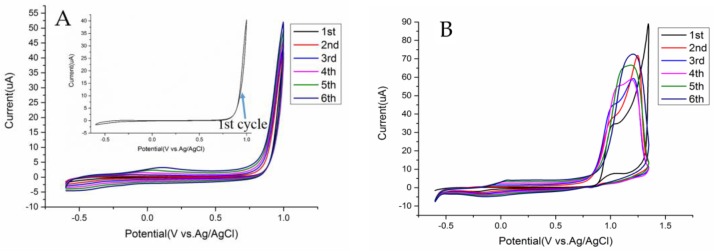
Six-repetitive cyclic voltammograms of 10.0 mM EDOT in aqueous solution containing the mixture of 0.1 M LiClO_4_ and 5 mM C_12_H_25_SO_4_Na in different potential range: −0.6 V–1.0 V (**A**); −0.6 V–1.35 V (**B**) Scan rate; 50 mV/s.

CV was selected for potentodynamic electropolymerization and it offered a continuous monitoring of the electroactivity of the polymer layer [23]. Then PEDOT films with different polymerization cycles, ranging from −0.6 V and 1.0 V, were fabricated and measured to optimize the most suitable deposition cycle. For comparison, one-step overoxidized PEDOT in the potential range from −0.6 V to 1.35 V was also prepared in different growth mechanism. In Figure 3A, an obvious current loop at the end of positive potential scan indicates the nucleation/growth mechanism for the formation of PEDOT [24], along with redox reaction of radical cations. The intensity of peaks at 0.125 V and −0.18 V increased with the growth of cycle number demonstrating the formation and continuous growth of PEDOT film on the Au microelectrode. Similar results had been reported earlier [25]. While the voltammograms in Figure 3B reveal that a large and actually irreversible oxidation peak appeared at 1.34 V with a high current value, as is observed in other PEDOT films [26]. The molecular mechanism has been illustrated in depth in a previous publication [27].

**Figure 4:**
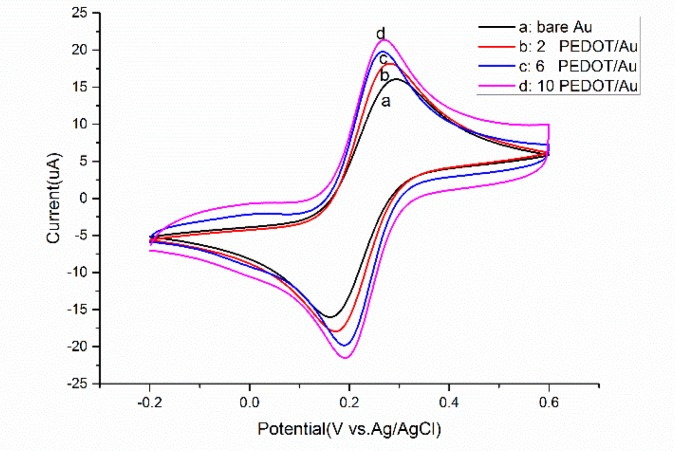
The cyclic voltammograms of different polymerization cycles prepared PEDOT/Au microelectrode in 5 mM [Fe(CN)_6_]^4−/3−^; scan rate: 100 mV/s.

**Figure 5:**
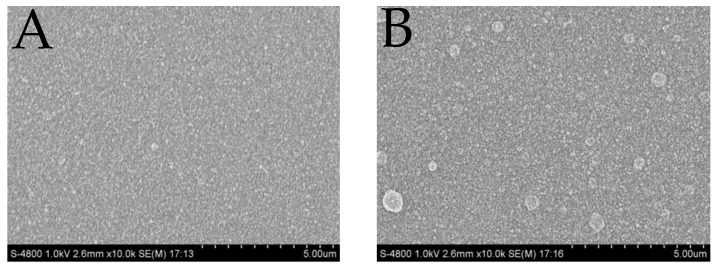
The scanning electron microscope (SEM) of 6 (**A**) and 10 (**B**) cycles PEDOT films with the magnification of 10 K and minimum scale of 500 nm.

A gradually increasing current with the cycles growing from two to 10 is shown in Figure 4, indicating the increase of the effective surface area of the PEDOT-modified microelectrode. The redox probe on 6-cycle PEDOT film exhibits the lowest oxidation potential, near 0.26 V, implying improved mass transfer properties between [Fe(CN)_6_]^3−/4−^ and polymers. In addition, Figure 5B shows small nuclei formation of thicker film after 10-cycle deposition which extremely affects the uniformity, adhesion and stability of the modified membrane. Thus six cycles was picked in the following overoxidation experiments.

### 3.3. The Characteristics of Films Under Different Overoxidation Time

**Figure 6:**
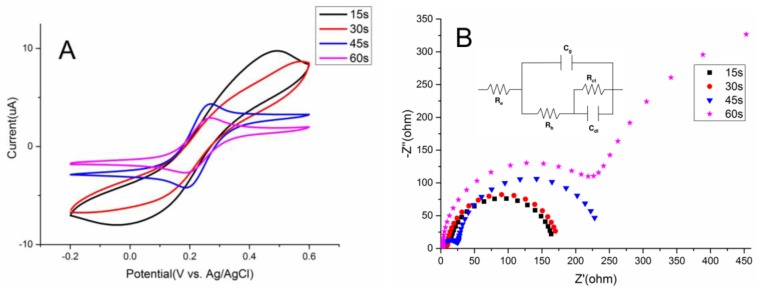
The cyclic voltammograms (**A**) and EIS spectrums (**B**) of 6-cycles PEDOT films with different overoxidation time in 5 mM [Fe(CN)_6_]^4−/3−^; scan rate: 100 mV/s.

The effect of overoxidation time on the electrical properties of PEDOT film was investigated with the assistance of CV and EIS in 0.1 M KCl containing 5 mM Fe (CN)_6_^4−/3−^. Figure 6A displays that no apparent redox peaks could be seen any more when PEDOT film was overoxidized for 15 s and 30 s at 1.35 V. However, when the duration lasted 45 s, a pair of well-defined redox peaks emerged probably because the film adsorbs ferricyanide and ferricyanide is transferred freely (or not limited) through the film which is in accordance with a previous report [11]. Thus it was an advantage in comparison with overoxidized PPy for its exclusion of anionic ferricyanide [28].

Furthermore, impedence spectra on the Au microelectrode (1 mm^2^) with different layers were evaluated using corresponding equivalent circuit (inset, Figure 6B). Fernández-Sánchez et al. concluded that the appearance at low frequency of a second semicircle (characterised by a second time constant) is fitted to a resistor/capacitor parallel combination in the equivalent circuit, namely a charge transfer resistance (Rct) and a double-layer capacitance (Cdl), respectively [29]. The calculated Rct values were 141.1 ohm·cm^2^ (15 s), 162.7 ohm·cm^2^ (30 s), 211.0 ohm·cm^2^ (45 s), 657.1 ohm·cm^2^ (60 s), respectively. As overoxidation time grew longer, the Rct value increased continuously. And the increment surged after 45 s overoxidation, from just 211.0 ohm·cm^2^ to 657.1 ohm·cm^2^. Moreover, the appearance of a second semicircle at low frequency response in curve c means some electrochemical adsorption occurred, and also the new electrode–electrolyte interface has already formed [29]. At the end of the optimization study, overoxidation condition was determined as 45 s at 1.35 V.

### 3.4. Comparisons Among Three Types of PEDOT Films: Oxidized State, One-Step Overoxidation and Double-Step Overoxidation

#### 3.4.1. Morphologies of the PEDOT Films on Au

**Figure 7:**
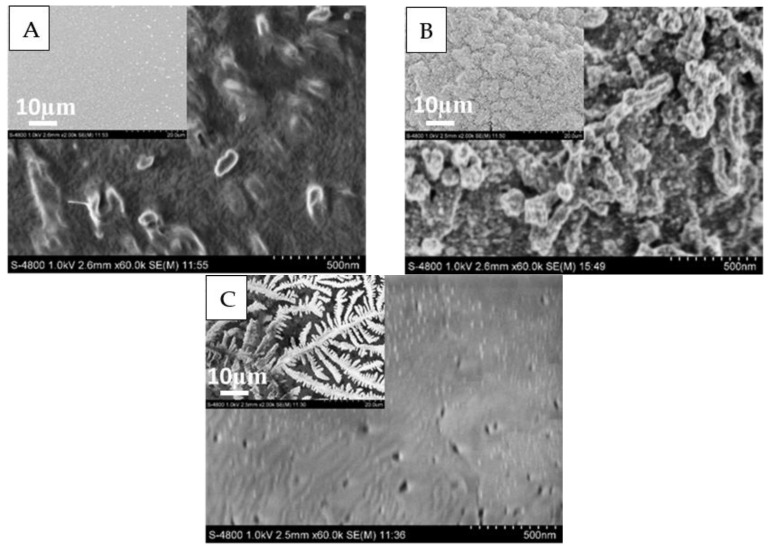
The SEM of PEDOT (**A**) one-step overoxidized PEDOT (**B**) and double-step overoxidized PEDOT (**C**) films with the magnification of 2 K and 60 K.

The morphologies of modified microelectrodes were investigated by SEM. As presented in Figure 7, image (A) shows a uniform film embedded with nodes, which indicated that PEDOT was in the oxidized (p-doped) state; image (B) shows a coarser surface coated with piles of spherical protrusions; image (C) shows a dendritic morphology yet with a nanoporous structure which is first reported here to the best of our knowledge. The appearance and growth of surface pores would allow the electrolyte to penetrate the polymer and eventually reach the electrode surface and spread over it. The noticeable morphology change implies that the overoxidation lasting 45 s at 1.35 V is harsh enough. This suggests an obviously increasing roughness upon overoxidation, presumably accompanying an inner porous structure. Actually when 1.35 V was applied on a 1 mm^2^ electrode, the initial current grew sharply up to 200 uA, which produced very high cell potential in the membrane, thus the rough topography and the more delicate node edges developed. This is in agreement with a previous study on poly(3-alkylthiophenes), demonstrating mechanical degradation of polymers at lower stress/strain [30].

#### 3.4.2. The EIS and FTIR of the PEDOT Films on Au

**Figure 8:**
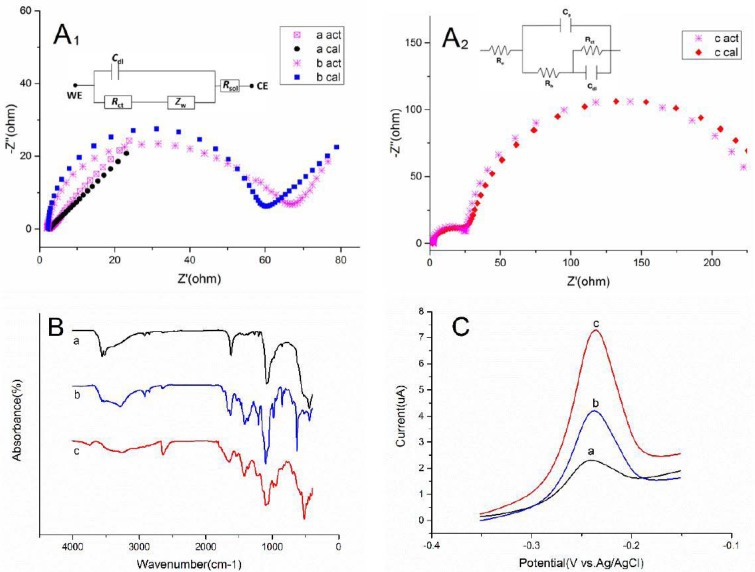
The EIS (**A_1_**,**A_2_**), FTIR (**B**) and DPSV (**C**) of PEDOT (a), one-step overoxidized PEDOT (b) and double-step overoxidized PEDOT (c) films.

EIS results on the Au microelectrode with different layers were fitted to two equivalent electrical circuits (inset, Figure 8A_1_,A_2_) [29,31], representing actual plots and calculated results. It is clear that PEDOT almost displayed a straight line with calculated Rct values 0.01 ohm·cm*^2^* (Figure 8A_1_, curve a) which is the characteristic of a diffusion-limited electrochemical process [32]. Apparently overoxidation obstructs electron transfer of the redox probe, with Rct values 54.33 ohm·cm*^2^* (curve b) and 211.0 ohm·cm^2^ (curve c). Comparatively speaking, double-step overoxidized PEDOT film in 5 mM [Fe(CN)_6_]^4−/3−^ exhibited similar conductivity as graphite electrode modified with 1,10-phenanthroline-5,6-dione in buffer solution [31]. Curve c shows two semicircles which suggests some electrochemical adsorption occurred [29]. Combined with porous surface structures in (Figure 7B), it is assumed that new electrode–electrolyte interface is already formed [29].

In order to identify the changes of functional groups between overoxidation, ATR-FTIR spectrum analysis was performed and quantified the same (Figure 8B). It seems that the disappearance of C–H bending mode near 890 cm^−1^ in the three spectra implies the formation of PEDOT chains with α,α’-coupling [33]. After overoxidation, curve b and curve c exhibit two strong bands at 1097/1099 cm^−1^ and 1205/1216 cm^−1^, ascribable to the symmetric and antisymmetric vibrations from the sulfone groups in the thiophene units, respectively [27]. Moreover the intense peak at 1668/1647 cm^−1^ is visible in curve b and curve c, probably illustrating the appearance of carbonyl groups. On the other hand, the broad signal at high wavenumbers upon overoxidation, is attributed to the stretching and bending vibrations in the hydroxyl moiety of the carboxylic acid group [14]. In contrast with curve a, new bands that appeared in the overoxidized materials at about 1415/1420 cm^−1^, originate from carboxyl symmetric stretching [34].

#### 3.4.3. The Electrochemical Response of the PEDOT Films on Au Microelectrodes to Lead Ion

The electrochemical responses of PEDOT film-modified Au microelectrodes were recorded with DPSV in 0.1 M HCl solution containing 2.4 uM Pb^2+^. 5 uL of Nafion ethanol solution (1 wt %) was then cast on the electrode surfaces and dried in air. For one thing, it acted as a binder immobilizing the modified polymer upon Au microelectrode; for another, its selective permeability succeeded to alleviate the interferences of anions [9].

From Figure 8C, all three types of PEDOT films show a stripping peak of lead ion at −0.242 V owing to the accumulation of Pb(II) on the pores of the polymer surfaces [35]. Also one can see that two different overoxidized films show higher current responses than PEDOT film. It is reported that polymers containing carboxyl groups were efficient adsorbents for lead ions [36], thus probably the complexation of Pb(II) with carboxyl groups in overoxidized PEDOT films is suggested, similar to the coordination complexes of Cu(II) ion with amino groups [37]. In addition, double-step overoxidized PEDOT films present the best sensing performance for lead ion in DPSV analysis due to its nanoporous structure and strong adsorption. To this extent, the double-step overoxidized PEDOT films can be fabricated for monitoring lead ion existing in the environment, food and biological samples.

## 4. Conclusions

According to the above experimental results, the initially high conductive and strongly adherent PEDOT films undergo morphological and electrochemical performance changes during overoxidation. The combination of SEM, CV, classical EIS and FTIR, provides a wealth of information about the process of overoxidation of thin PEDOT films such as coarser surface morphology, well-refined redox response, newly formed electrode–electrolyte interface formation and abundant oxygen functional groups. During DPSV measurements, double-step overoxidized film shows the highest affinity toward lead ion. It is also surmised that double-step overoxidized PEDOT film can be used to study adsorption and desorption characteristics of ions or bioorganic molecules in the future.
